# Highly Purified Eicosapentaenoic Acid Alleviates the Inflammatory Response and Oxidative Stress in Macrophages during Atherosclerosis via the miR-1a-3p/sFRP1/Wnt/PCP-JNK Pathway

**DOI:** 10.1155/2022/9451058

**Published:** 2022-04-13

**Authors:** Tongtong Zang, Han Chen, Shutong Shen, Fei Xu, Rui Wang, Jia Yin, Xiehui Chen, Min Guan, Li Shen, Haobo Pan, Junbo Ge

**Affiliations:** ^1^Department of Cardiology, Zhongshan Hospital, Fudan University, Research Unit of Cardiovascular Techniques and Devices, Chinese Academy of Medical Sciences, Shanghai, China; ^2^Shanghai Institute of Cardiovascular Diseases, Shanghai, China; ^3^National Clinical Research Center for Interventional Medicine & Shanghai Clinical Research Center for Interventional Medicine, Shanghai, China; ^4^Research Center for Human Tissue and Organs Degeneration, Institute of Biomedicine and Biotechnology, Shenzhen Institutes of Advanced Technology, Chinese Academy of Sciences, Shenzhen, China; ^5^Shenzhen Longhua District Central Hospital/the Affiliated Central Hospital of Shenzhen Longhua District, Guangdong Medical University, Shenzhen, China

## Abstract

Highly purified eicosapentaenoic acid (EPA) has shown great effects in the prevention of atherosclerosis. In a murine model, it significantly reduced plaque accumulation, lowered plasma lipid levels, and decreased inflammation levels, which was also observed *in vitro*. Using microRNA sequencing, we identified differentially expressed microRNAs, among which miR-1a-3p was selected for further validation. Overexpression of miR-1a-3p in RAW264.7 cells worsened lipid accumulation, increased oxidative stress, and exacerbated inflammatory responses whereas its downregulation produced the opposite results. Potential targets of miR-1a-3p were analyzed by prediction tools. Then, secreted frizzled-related protein 1 (sFRP1), an antagonist of the Wnt pathway, was confirmed as the target gene of miR-1a-3p by a dual-luciferase reporter assay. Further research showed that in macrophages, EPA influenced the activation of the Wnt/planar cell polarity-c-Jun N-terminal kinase (Wnt/PCP-JNK) axis, which is consistent with the phenomenon that miR-1a-3p has an impact on this same axis. Collectively, our findings suggest that EPA mitigates inflammatory responses and oxidative responses both *in vivo* and *in vitro* by targeting the miR-1a-3p/sFRP1/Wnt/PCP-JNK axis in macrophages, which may explain the cardioprotective role of EPA and promote the application of EPA in clinical practice.

## 1. Introduction

Atherosclerosis is an independent risk factor for stroke and myocardial infarction, leading to severe outcomes. Inflammation plays an important role in this process. Repeated cycles of inflammation lead to the accumulation of macrophages, which secrete cytokines and increase the contents of macrophages, worsening this vicious cycle. Increased inflammation induces vascular smooth muscle cell proliferation and migration in lesions, leading to plaque formation [1]. Inflammation also leads to endothelial dysfunction, disrupting normal vascular mobility and accelerating the course of atherosclerosis [2]. Omega-3 polyunsaturated fatty acids (PUFAs), including docosahexaenoic acid (DHA) and eicosapentaenoic acid (EPA), have a wide range of applications against cardiovascular diseases. Here, we mainly focused on EPA. Previous studies have shown that EPA exerts an anti-inflammatory role in atherosclerosis. Recently, the positive results from the Reduction of Cardiovascular Events with Icosapent Ethyl-Intervention Trial (REDUCE-IT) renewed hope for highly purified EPA [3], urging us to investigate its mechanisms of action. Among the pathways related to inflammation, Wnt is an essential evolutionarily conserved pathway. There have been studies involving EPA and the canonical Wnt pathway [4]. However, the noncanonical Wnt/planar cell polarity-c-Jun N-terminal kinase (Wnt/PCP-JNK) pathway is more closely related to oxidative stress and inflammation, which are important parts of atherosclerosis. Based on these facts, we hypothesize that EPA may also influence Wnt/PCP-JNK, the noncanonical Wnt pathway.

MicroRNAs are noncoding single-stranded RNAs encoded by endogenous genes. In atherosclerosis, microRNAs participate in lipid metabolism, inflammation, and phenotype translation [5–8]. EPA can influence a wide range of diseases through microRNAs, such as brown thermogenesis, tumorigenesis, and tumor metastasis [9–12]. However, the relationship between EPA and microRNAs in atherosclerosis is unclear. If we can determine how EPA works via microRNAs in atherosclerosis, we may be able to explain the mechanism for the direct utilization of EPA in clinical practice.

In the current study, we prepared highly purified EPA (no less than 97%) and examined its antiatherosclerotic effects in a murine model and macrophages. We also explored whether and how EPA alleviated inflammatory responses and oxidative stress in macrophages by microRNA sequencing and bioinformatics analysis. Then, we targeted miR-1a-3p/secreted frizzled-related protein (sFRP1). Since sFRP1 is an antagonist of the Wnt pathway and Wnt/PCP-JNK is closely related to inflammation, we hypothesized that EPA may act via the miR-1a-3p/sFRP1/Wnt/PCP-JNK pathway.

## 2. Materials and Methods

### 2.1. Gas Chromatography

The purified EPA sample was evaporated to form a gas and injected into the top of the chromatographic column, where the purified EPA vapor was carried into the column by an inert gas. Then, the component was identified and quantified following the GB 5009.168-2016-Method 3-Normalization Method. Both low-purity fish oil soft gels and high-purity fish oil soft gels were purchased from the pharmacy.

### 2.2. Animal Experiments

**Figure 1:**
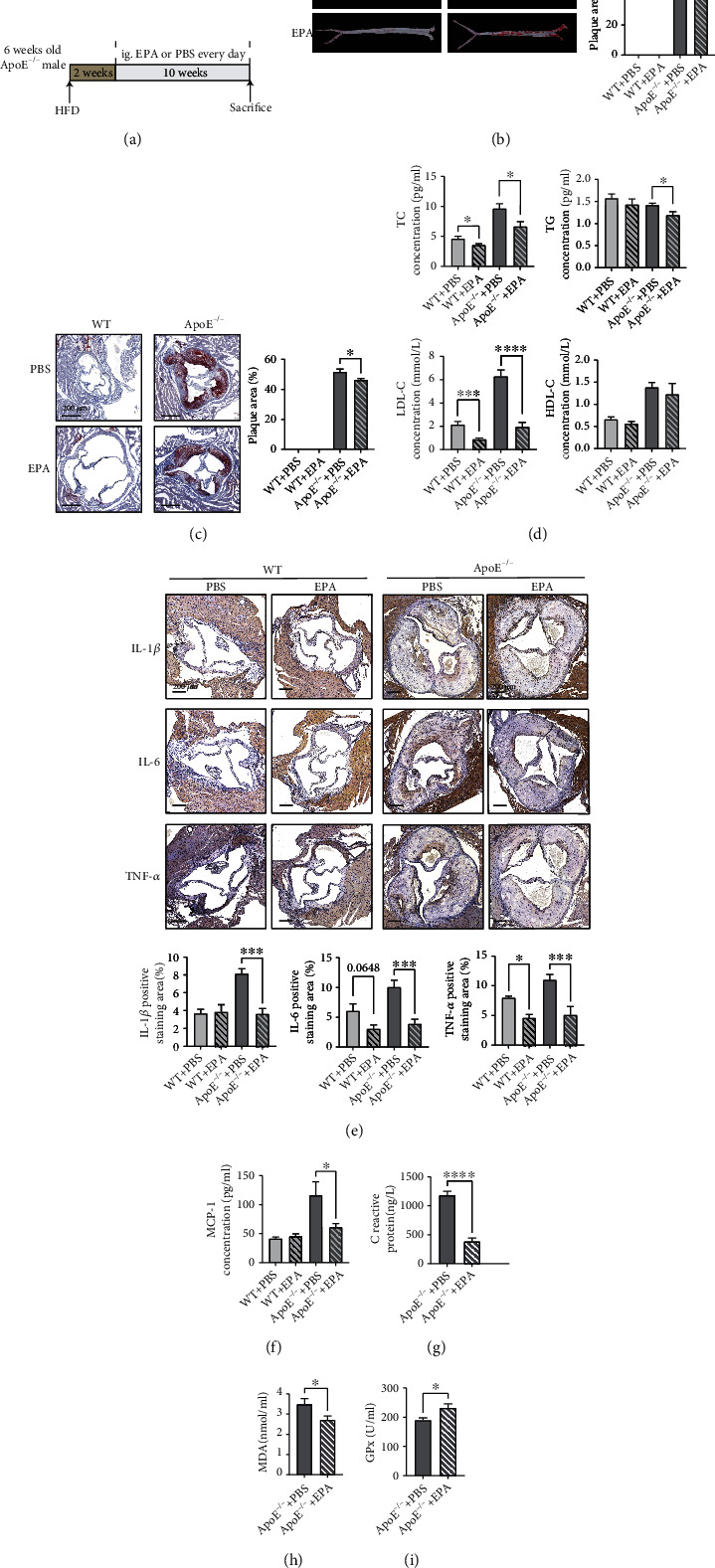
EPA exerts antiatherosclerotic and anti-inflammatory effects *in vivo*. (a) Schematic depiction of the study in which ApoE^−/−^ mice were fed a HFD for 12 weeks and treated with PBS or EPA starting at week 3. (b) Gross ORO staining of the aortas (*n* = 5 per group). (c) Aortic slices after ORO staining of mice treated with EPA (*n* = 5 per group). (d) Serological TC, TG, LDL-C, and HDL-C levels (*n* = 6-9). (e) IHC of mouse aortic roots: IL-1*β*, IL-6, and TNF-*α* levels (*n* = 5 per group). (f) Serological MCP-1 levels (*n* = 5-9). (g) Serological CRP level (*n* = 10-14). (h) Serological level of MDA (*n* = 7-9). (i) Serological level of GPx (*n* = 5 per group). The data are presented as the mean ± SEM. ^∗^*p* < 0.05,  ^∗∗^*p* < 0.01, and^∗∗∗^*p* < 0.001.

The Ethics Committee of Zhongshan Hospital, Fudan University, approved all animal experiments, which were conducted in the animal laboratory of Zhongshan Hospital, Fudan University (Shanghai, China). Six-week-old male apolipoprotein E-deficient (ApoE^−/−^) mice on a C57BL/6 background and wild-type (WT) C57BL/6 mice were purchased from GemPharmatech (China). All animals were given a high-fat diet (HFD) with 40 kcal% fat, 1.25% cholesterol, and 0.5% cholic acid (Research Diets, United States) for 12 weeks. After 2 weeks of being fed a HFD, both ApoE^−/−^ mice and WT mice were randomly distributed into a PBS treatment (daily, 0.1 ml, ig) or EPA treatment (daily, 0.6 mg/g, ig) group for 10 weeks of intervention (Figure 1(a)). The dose of EPA was converted from the human dose in the REDUCE-IT trial [3].

### 2.3. Cell Culture

The RAW264.7 cell line was purchased from Shanghai Zhong Qiao Xin Zhou Biotechnology Co., Ltd. (China), and cultured in DMEM (Gibco, United States) with 10% fetal bovine serum (Gibco, United States) with penicillin/streptomycin solution (HyClone, United States). For foam cell induction, cells were incubated with 50 mg/ml ox-LDL (Yiyuan Biotech, China) for 24 h. Purified EPA was dissolved in DMSO and then diluted with culture media to reach the desired concentration.

### 2.4. Quantification of SOD, GPx, and MDA

The levels of oxidative markers SOD (Superoxide Dismutase), GPx (Glutathione peroxidase), and MDA (Malondialdehyde) in serum and cell lysate were determined by the commercial SOD assay kit (Beyotime, Shanghai, China), GPx assay kit (Beyotime, Shanghai, China), and MDA assy kit(Beyotime, Shanghai, China), according to the respective manufacturer's protocols.

### 2.5. The Detection of CRP Level

The level of CRP (C-reactive protein) was determined by the commercial CRP assay kit (Multi science, China) according to the manufacturer's protocol.

### 2.6. Cell Counting Kit 8 (CCK8)

Approximately 100 *μ*l (2000) of RAW264.7 cells were added to 96-well plates. After ox-LDL + EPA treatment, the medium in the plates was removed. Then, 10 *μ*l of CCK8 solution (Beyotime, China) and 90 *μ*l of medium were added to each well. Incubation continued in a cell culture incubator for 0.5 hours. The absorbance of each well was measured at 450 nm with an Epoch™ 2 microplate spectrophotometer (BioTek, United States) following the manufacturer's instructions. Wells with the appropriate amounts of cell culture medium, EPA, and CCK8 solution but not cells were used as blank controls.

### 2.7. Total RNA/miRNA Isolation

Total RNA was isolated and purified from cells with TRIzol reagent (Invitrogen, United States). The miRcute miRNA Isolation Kit (Tiangen, China) was used to isolate miRNA. The concentrations and purities of the RNA samples were determined with a NanoDrop 2000 (Thermo Fisher Scientific, United States).

### 2.8. miRNA Library Construction, Sequencing, and Data Analyses

A total RNA Purification Kit (LC Sciences, Houston, United States) was used to isolate and purify total RNA from samples according to the protocol provided by the manufacturer. RNA integrity was then examined with a Bioanalyzer 2100 (Agilent, CA, United States) at concentrations > 50 ng/*μ*l. Approximately 1 *μ*g of total RNA was used in the miRNA sequencing assay. TruSeq Small RNA Sample Prep Kits (Illumina, San Diego, United States) were used to construct small RNA libraries according to the kit instructions. A High Sensitivity DNA Chip Kit (Agilent, CA, United States) was used to process the library quality control. Then, we performed single-end sequencing (1 × 50 bp) on an Illumina HiSeq 2500 (LC Bio, China) following the vendor's recommended protocol.

### 2.9. Reverse Transcription and Quantitative Real-Time PCR (RT–qPCR)

Reverse transcription and quantitative real-time PCR of miRNA were conducted with a miRcute Plus miRNA First-Strand cDNA Kit (Tiangen, China) and miRcute Plus miRNA qPCR Kit (Tiangen, China). Reverse transcription and quantitative real-time PCR of mRNA were performed with a PrimeScript™ RT reagent Kit with gDNA Eraser (TAKARA, China) and SYBR Premix Ex Taq™ (TAKARA, China). All procedures were performed according to the manufacturer's recommended protocol. The sequences of the PCR primers used in this study are shown in Table S1.

### 2.10. Transfection of miRNA Mimics/Inhibitors and Plasmids

miR-1a-3p mimics, inhibitors, and negative controls (NCs) were purchased from GenePharma (China). Transfection of the miRNA mimics, inhibitors, and negative controls was performed with Lipofectamine™ 3000 transfection reagent (Thermo Fisher Scientific, United States) following the manufacturer's instructions. Transfection of the sFRP1 plasmid was performed with Lipofectamine™ 3000 transfection reagent (Thermo Fisher Scientific, United States) and P3000. After 6 hours of transfection, the medium was replaced.

### 2.11. Oil Red O Staining

Oil Red O staining was conducted on both cells and tissues. After 24 hours of treatment with EPA and a subsequent 24 hours of incubation with ox-LDL, RAW264.7 cells were subjected to oil red O staining (ORO). To investigate the effects of miRNA on lipid accumulation, ORO staining was conducted after 24 hours of treatment with miRNA mimics, inhibitors or NCs and a subsequent 24 hours of incubation with ox-LDL. The cells were washed with PBS and then fixed with 4% paraformaldehyde (PFA) for 30 min at room temperature. Next, the cells were incubated with ORO staining solution (Sangon Biotech, China) filtered through a strainer (0.45 *μ*m) for 90 min. Finally, the cells were processed for hematoxylin staining for 3 min at room temperature. To stain the mouse aortic roots, mouse aortic root tissues were immediately snap-frozen in lipid nitrogen after sacrifice. Then, the tissues were placed in OCT cryostat embedding compound (Tissue-Tek, United States) and sectioned. The mouse aortic root sections were stained with ORO staining solution. A bright-field microscope (Leica, Germany) was utilized to observe the lipid droplets.

### 2.12. Western Blot

After treatment, cells were lysed with cold RIPA lysis buffer (Beyotime, China) according to the manufacturer's instructions. The protein concentrations were determined with a BCA Protein Assay kit (Beyotime, China). Proteins were separated by SDS–PAGE, transferred to nitrocellulose filter membranes (Millipore, United States), blocked in 5% BSA in TBST for 2 h, and incubated overnight with primary antibodies at 4°C. The primary antibodies and dilutions were as follows: anti-GAPDH (1 : 2000; Cell Signaling Technology, #5147, United States), anti-IL-1*β* (1 : 1000; Abcam, #ab234437, United States), anti-NLRP3 (1 : 1000; Abcam, #ab263899, United States), anti-sFRP1 (1 : 1000; Abcam, #ab4139, United States), anti-JNK (1 : 1000; Cell Signaling Technology, #9252, United States), and anti-phospho-JNK (1 : 1000; Cell Signaling Technology, #4668, United States). Next, nitrocellulose filter membranes were exposed to horseradish peroxidase- (HRP-) conjugated secondary antibodies (Cell Signaling Technology, United States). Luminata™ Forte Western HRP Substrate (Millipore, United States) was used to detect the signals.

### 2.13. Immunohistochemistry (IHC)

Immunohistochemistry of tissue sections was performed as described previously [13]. Cell nuclei were stained with hematoxylin (Servicebio, Wuhan, China). The primary antibodies and dilutions were as follows: anti-IL-1*β* (1 : 200; Abcam, #205924, United States), anti-IL-6 (1 : 200; Abcam, #208113, United States), and anti-TNF-*α* (1 : 200; Abcam, #1763, United States). The secondary HRP-labeled antibody of the corresponding species was purchased from Servicebio (Wuhan, China).

### 2.14. Flow Cytometry Analysis of Reactive Oxygen Species

We used DCFH-DA (Sigma, United States) to measure the intracellular ROS production. To analyze the ROS levels in cells, RAW264.7 cells were seeded in 12-well plates. After 24 hours of treatment with EPA or miR-1a-3p and a subsequent 24 hours of incubation with ox-LDL, the cells were washed with sterile PBS, and 5 *μ*M DCFH-DA in DMEM was added to the wells. Then, the cells were incubated at 37°C for 30 min and washed again. Finally, the cells were digested, and the ROS levels were detected by flow cytometry (BD Biosciences, San Jose, CA, United States). For FCM detection, each group was tested in triplicate. The data obtained from FCM were analyzed with FlowJo software (Tree Star Inc., Ashland, OR, United States).

### 2.15. Dual-Luciferase Reporter Assays

For reporter assay analyses, 2 × 10^4^ HEK293T cells were plated in a 96-well plate. Then, cells were transfected with 50 nM miR-1a-3p or the mimic NC (Hanbio Biotechnology, China). The cells were then cotransfected with 2 *μ*g/mL vector expressing the wild-type or mutant sFRP1 3′-UTR. After 48 h, a Dual-Luciferase Reporter Assay System (Promega, Madison, WI, United States) was used to measure luciferase activity based on the manufacturer's instructions.

### 2.16. Statistical Analysis

The measured data were normally distributed. The results are expressed as the means ± standard error of the mean (SEM). Comparisons among two groups were performed using Student's *t*-test. Comparisons among multiple groups were performed using one-way analysis of variance (ANOVA) followed by Dunnett's multiple comparisons test for multiple comparisons. Additionally, *P* < 0.05 was considered to indicate a significant difference.

## 3. Results

### 3.1. EPA Alleviated Atherosclerotic Plaque Formation and Reduced Inflammatory Responses in *ApoE*^−/−^ Mice

The EPA in our research was highly purified, and its gas chromatography results are shown in Table S2. Comparison of our purified EPA to fish oil soft gels sold on the market showed that the EPA in our research had a very high purity. The impurities (1%) were *α*-linolenic acid (ALA) and *γ*-linolenic acid (GLA), two important unsaturated fatty acids. Therefore, the EPA used in our research could be thought of as a pure unsaturated fatty acid.

To explore the effects of EPA on the atherosclerotic process, we detected the aortic conditions of mice. Gross oil red O (ORO) staining showed that plaque formation in EPA-treated *ApoE*^−/−^ mice was approximately 10% lower than that in PBS-treated *ApoE*^−/−^ mice (Figure 1(b)). ORO staining of the aortic roots showed that mice treated with EPA had smaller plaque areas (Figure 1(c)). Apart from triglycerides (TGs) and total cholesterol (TC), LDL cholesterol (LDL-C) and HDL cholesterol (HDL-C) metabolism has been highlighted in the atherosclerotic process [14, 15]. Although the HDL-C levels remained almost unchanged, EPA treatment downregulated the TC, TG, and LDL-C contents, which improved hyperlipidemia and reduced atherosclerosis (Figure 1(d)).

Previous studies have also revealed that EPA displays strong anti-inflammatory effects in cardiovascular diseases [16]. Here, we mainly focused on the effects of EPA on atherosclerosis. In our research, EPA lowered monocyte chemotactic protein-1 (MCP-1)/chemokine (C-C motif) ligand 2 (CCL-2) levels in serum, which means that EPA could attenuate inflammatory responses during atherosclerosis (Figure 1(f)). We also detected the level of CRP (C-reactive protein) in *ApoE*^−/−^+PBS and *ApoE*^−/−^+EPA group to show the effects of EPA in atherosclerosis. The result suggested that EPA significantly reduce the level of CRP (Figure 1(g)), which is consistent with the result in REDUCE-IT trial [3]. The levels of IL-1*β*, IL-6, and TNF-*α* were evaluated in the aortas of mice. IHC showed that EPA reduced IL-1*β*, IL-6, and TNF-*α* levels in plaque areas to ameliorate inflammation (Figure 1(e)).

Oxidative stress is an important driving factor in atherosclerosis. We detected the level of oxidative stress makers SOD (Superoxide Dismutase), GPx (Glutathione peroxidase), and MDA (Malondialdehyde) in the serum of atherosclerotic mice. The results showed that EPA can reduce the level of MDA and improve the level of GPx (Figures 1(h) and 1(i)). The level of SOD showed a trend of decreasing but did not reach statistical significance (Figure S1). All suggested a downregulation of oxidative stress in mice.

Overall, EPA relieved plaque progression, lowered blood lipid levels, and decreased inflammatory responses in both serum and aortic roots, which means that EPA shows significant antiatherosclerotic effects in a murine model.

### 3.2. EPA Attenuated Lipid Accumulation, Reduced Inflammatory Responses, and Lowered Oxidative Stress in Macrophages in a Dose-Dependent Manner

**Figure 2:**
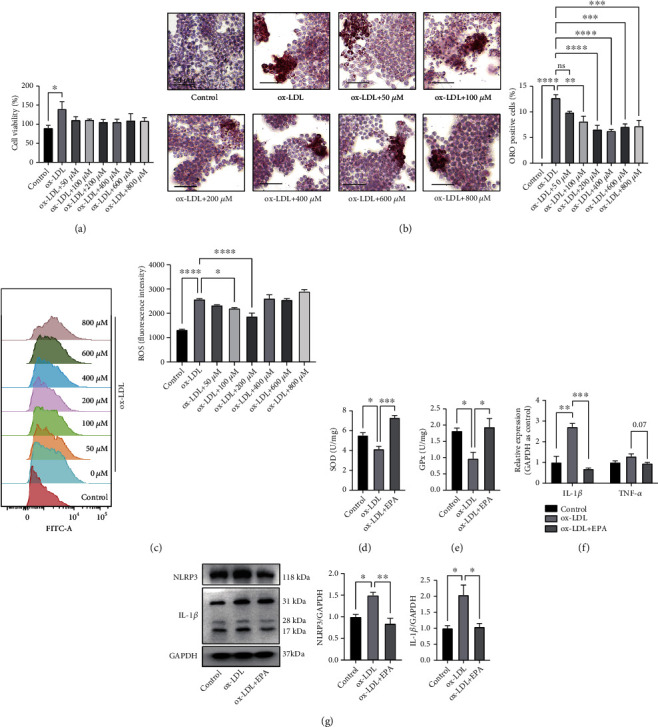
EPA reduced lipid accumulation, inflammatory responses and oxidative stress in macrophages. (a) CCK8 of macrophages treated with ox-LDL and different concentrations of EPA (0 *μ*M, 50 *μ*M, 100 *μ*M, 200 *μ*M, 400 *μ*M, 600 *μ*M, and 800 *μ*M) (*n* = 3 each group). (b) Representative image of ORO staining and the percentage of foam cells. Cells were treated with ox-LDL and different concentrations of EPA (0 *μ*M, 50 *μ*M, 100 *μ*M, 200 *μ*M, 400 *μ*M, 600 *μ*M, and 800 *μ*M). Approximately 1000 cells were counted per treatment over six separate experiments. (c) The intracellular ROS levels in macrophages treated with ox-LDL and different concentrations of EPA (0 *μ*M, 50 *μ*M, 100 *μ*M, 200 *μ*M, 400 *μ*M, 600 *μ*M, and 800 *μ*M) detected by flow cytometry (*n* = 3 per group). (d) The level of SOD in macrophages treated with ox-LDL and ox-LDL + 200 *μ*M EPA treatment (*n* = 3 per group). (e) The level of GPx in macrophages treated with ox-LDL and ox-LDL + 200 *μ*M EPA treatment (*n* = 3 per group). (f) mRNA levels of IL-1*β* and TNF-*α* after ox-LDL treatment and ox-LDL + 200 *μ*M EPA treatment evaluated by qRT–PCR and normalized to GAPDH (*n* = 3 per group). (g) WB results of NLRP3 and IL-1*β* after ox-LDL treatment and ox-LDL + 200 *μ*M EPA treatment (*n* = 3 per group). The data are presented as the mean ± SEM. ^∗^*p* < 0.05,  ^∗∗^*p* < 0.01, and^∗∗∗^*p* < 0.001.

Macrophages serve as the initiators of atherosclerosis throughout the whole process of lipid formation and the inflammatory response. Combining the fact that EPA acts via anti-inflammation and our *in vivo* results, we next focused on the effects of EPA in macrophages. Before researching the effects and mechanism of EPA on macrophages, we examined the effects of EPA on the proliferation and apoptosis of ox-LDL-treated macrophages. The CCK8 results showed that 50 mg/ml ox-LDL significantly promoted the proliferation of macrophages and 50 *μ*M, 100 *μ*M, 200 *μ*M, 400 *μ*M, 600 *μ*M, and 800 *μ*M EPA could curb this trend (Figure 2(a)). RAW264.7 cells incubated with ox-LDL showed notable positive oil red O staining, while treatment with a gradient of concentrations of EPA reversed this trend to some extent (Figure 2(b)). In the cases where more than 200 *μ*M EPA was used, the effects did not increase even if the drug concentration increased. Previous studies showed that the effects of EPA were closely related to concentration. Different concentrations may lead to opposite results [17]. It may be explained that excess EPA may lead to the inhibition of Na, K-ATPase, causing cell dysfunction [18, 19]. All these side effects may lead to ROS production and counteract the beneficial effects. Reactive oxygen species (ROS) participate in all phases of plaque formation and rupture. Excessive ROS can lead to a decrease in plaque stability and the activation of inflammatory mediators [20]. In addition, almost all cardiovascular risk factors, including smoking, hypercholesterolemia, hypertension, and diabetes mellitus, can increase ROS levels [21]. In RAW264.7 cells, flow cytometry analysis showed that ox-LDL treatment induced excessive ROS production, while EPA reversed this trend (Figure 2(c)) and 200 *μ*M EPA showed the best effects. Combining the CCK8, ROS, and ORO staining results, we chose 200 *μ*M EPA as our concentration for subsequent experiments. Then, we detected the levels of SOD and GPx to explore the change of oxidative stress markers after EPA treatment. The results showed that ox-LDL treatment reduced the level of SOD and GPx, which is consistent with the results that ROS production was increased. The addition of 200 *μ*M EPA could significantly reverse the trends (Figures 2(d) and 2(e)).

In terms of inflammation, the levels of IL-1*β* and TNF-*α* were tested in RAW264.7 cells. The results showed that the mRNA levels of IL-1*β* and TNF-*α* were increased after incubation with ox-LDL and decreased after treatment with 200 *μ*M EPA (Figure 2(f)). NLRP3 activates caspase-1, which cleaves pro-IL-1*β* into IL-1*β*, leading to downstream inflammatory cytokine release and macrophage activation [22]. We identified NLRP3 and IL-1*β* as two important inflammatory markers. As our results show, NLRP3 and IL-1*β* increased after ox-LDL induction and were suppressed by 200 *μ*M EPA treatment (Figure 2(g)), which is consistent with the *in vivo* results that EPA can lower the inflammatory response.

### 3.3. MicroRNA Sequencing Was Utilized to Identify Differentially Expressed MicroRNAs in Microphages

The role of microRNAs in cardiovascular diseases has recently been recognized and emphasized [23–25]. EPA (200 *μ*M) showed the best effects in the ORO staining and inhibition of ROS generation assays. Therefore, RNA from RAW264.7 cells treated with ox-LDL + DMSO or ox-LDL + 200 *μ*M EPA was extracted, and microRNA sequencing was conducted to identify differentially expressed microRNAs.

**Figure 3:**
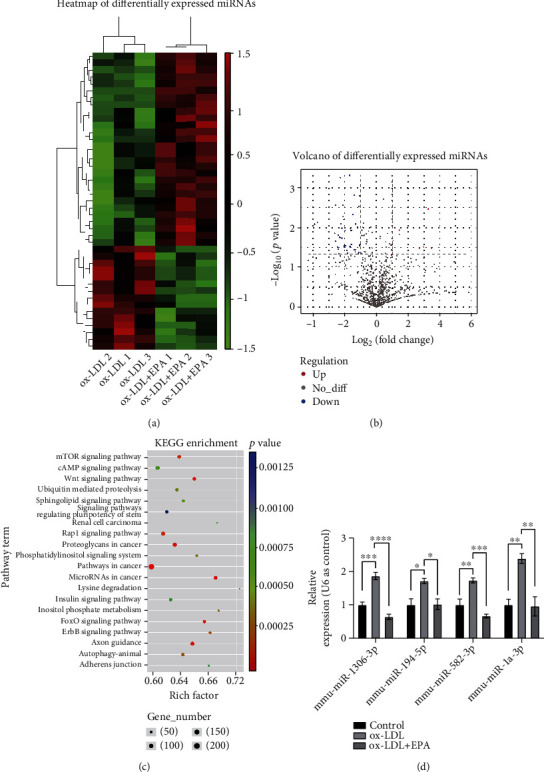
MicroRNA sequencing revealed that miR-1a-3p was a participant after EPA treatment. (a) Heatmap showing the differentially expressed microRNAs. (b) Volcano map showing upregulated and downregulated microRNAs. (c) KEGG analyses of the 10 most changed microRNAs. (d) qRT–PCR of the top changed miRNAs normalized to U6. The data are presented as the mean ± SEM. ^∗^*p* < 0.05,  ^∗∗^*p* < 0.01, and^∗∗∗^*p* < 0.001.

**Figure 4:**
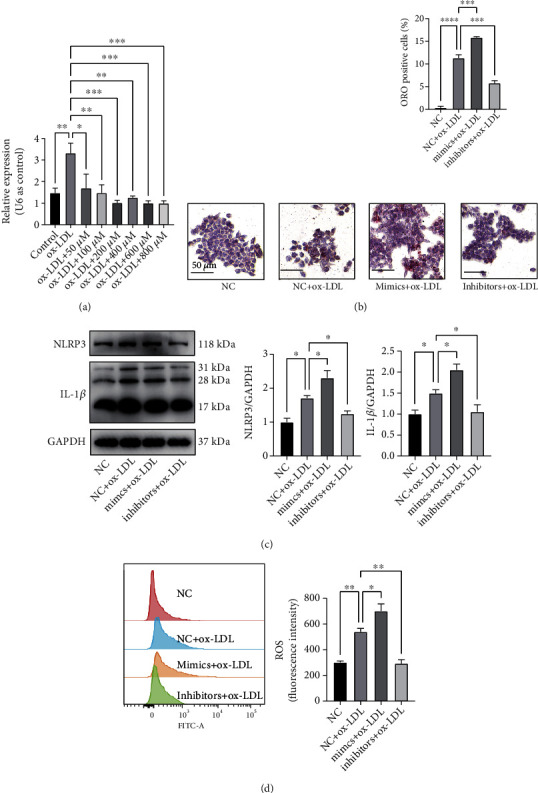
miR-1a-3p mediated atherosclerotic process *in vitro*. (a) The relative expression of miR-1a-3p after treatment with ox-LDL and different concentrations of EPA (0 *μ*M, 50 *μ*M, 100 *μ*M, 200 *μ*M, 400 *μ*M, 600 *μ*M, and 800 *μ*M) evaluated by qRT–PCR (normalized to U6) (*n* = 3 per group). (b) Representative images of ORO staining and the percentage of foam cells, showing that miR-1a-3p mediated foam cell formation. Approximately 1000 cells were counted per treatment over six separate experiments. (c) WB results of NLRP3 and IL-1*β* after ox-LDL treatment and miR-1a-3p mimic/inhibitor transfection (*n* = 3 per group). (d) The intracellular ROS levels detected by flow cytometry after ox-LDL treatment and miR-1a-3p mimic/inhibitor transfection (*n* = 3 per group). (e) The level of GPx in macrophages treated with ox-LDL and ox-LDL + 200 *μ*M EPA treatment (*n* = 3 per group). The data are presented as the mean ± SEM. ^∗^*p* < 0.05,  ^∗∗^*p* < 0.01, and^∗∗∗^*p* < 0.001.

Based on the microRNA sequencing results, 15 microRNAs were upregulated and 28 microRNAs were downregulated (*p* < 0.05) (Figures 3(a) and 3(b)). Kyoto Encyclopedia of Genes and Genomes (KEGG) analyses of the 10 most changed microRNAs showed that the differentially expressed genes (DEGs) were enriched in several vital pathways during EPA treatment, of which the Wnt signaling pathway changed the most (Figure 3(c)). Sorted by *p* value, the top microRNAs were validated. Based on the qPCR result, mmu-miR-1306-3p, mmu-582-3p, and miR-1a-3p were selected for further study. However, the expression of miR-582-3p was quite low and the research of miR-582-3p was rarely related to inflammation or oxidative stress. It was mainly about organ development and morphology [26]. But there have been research linking oxidative stress and inflammation with miR-1306-3p and miR-1a-3p [27, 28]. Therefore, we transfected these two microRNAs NC/mimics/inhibitors into cells. However, the interference of miR-1306-3p did not affect the lipid accumulation in macrophages (Fig. S3) while the interference of miR-1a-3p significantly influenced the lipid accumulation (Figure 4(b)). miR-1a-3p was upregulated after ox-LDL treatment and downregulated when EPA was added (Figure 3(d)). Thus, miR-1a-3p was chosen for further research.

### 3.4. miR-1a-3p Participated in the Processes of Lipid Accumulation, the Inflammatory Response, and Oxidative Stress

Next, the potential role of miR-1a-3p in atherosclerosis was explored. First, we examined the inhibitory effects of EPA on miR-1a-3p. The results showed that 50 *μ*M, 100 *μ*M, 200 *μ*M, 400 *μ*M, 600 *μ*M, and 800 *μ*M EPA could significantly lower the level of miR-1a-3p. The trend was that as the EPA concentration increased, the miR-1a-3p level decreased (Figure 4(a)). Then, miR-1a-3p mimics and miR-1a-3p inhibitors were transfected into RAW264.7 cells. In cells incubated with ox-LDL, a positive oil red staining was confirmed (Figure 4(b)). Transfection of miR-1a-3p mimics significantly aggravated lipid accumulation, while transfection of miR-1a-3p inhibitors displayed the opposite result. Western blotting analysis showed that compared to the NC group, the miR-1a-3p mimics enhanced the protein levels of NLRP3 and IL-1*β*, causing the downstream release of cytokines. In contrast, miR-1a-3p inhibitors alleviated the inflammatory response, diminished the protein levels of NLRP3 and IL-1*β*, and ameliorated the downstream inflammatory activation of cytokines (Figure 4(c)).

A similar trend was detected in the ROS levels by flow cytometry. Compared with NC-transfected and ox-LDL-treated cells, miR-1a-3p mimics augmented ROS generation and decreased the level of GPx, leading to severe oxidative stress. In contrast, miR-1a-3p inhibitors attenuated ROS generation and increased the level of GPx, reducing oxidative stress (Figures 4(d) and 4(e)). The interference of miR-1a-3p did not influence the level of SOD (Fig. S2).

### 3.5. miR-1a-3p Regulated Atherosclerosis via the miR-1a-3p/sFRP1/Wnt/PCP-JNK Axis

**Figure 5:**
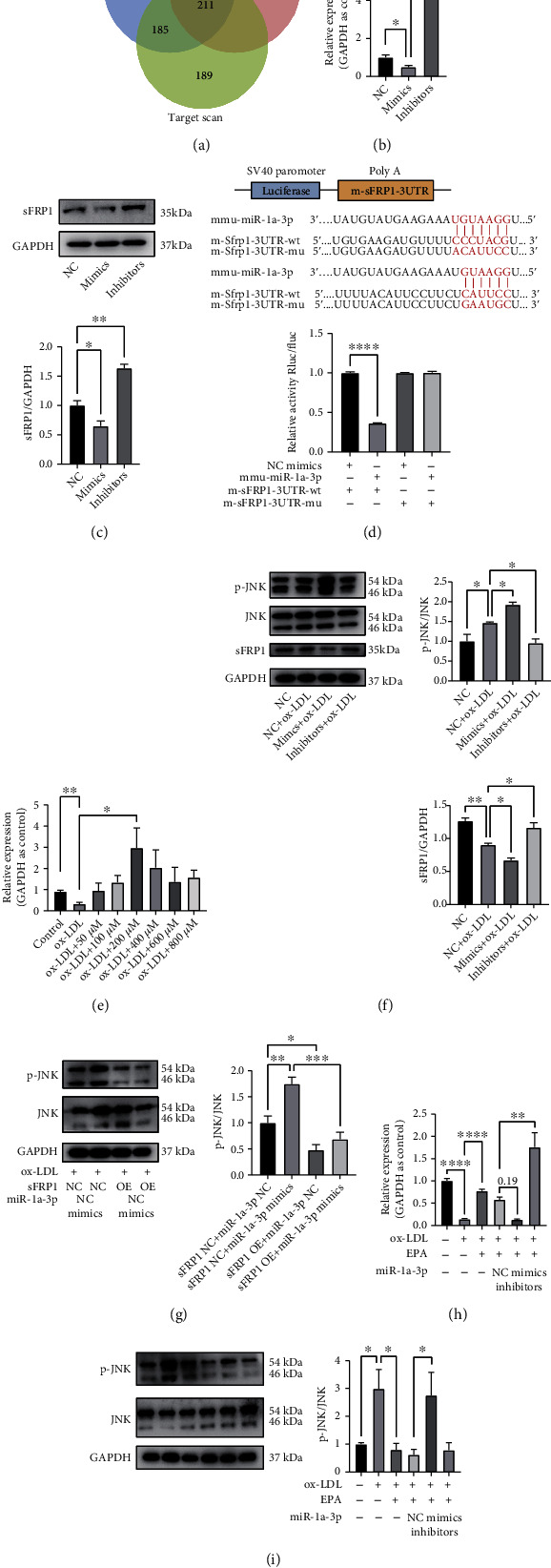
miR-1a-3p participated in the atherosclerotic process through the sFRP1/Wnt/PCP-JNK axis. (a) The results from the online prediction tools for miR-1a-3p. (b) The mRNA levels of sFRP1 after NC and miR-1a-3p mimic/inhibitor transfection (*n* = 3 per group). (c) WB analyses of sFRP1 after NC and miR-1a-3p mimic/inhibitor transfection (*n* = 3 per group). (d) Sequence alignment of miR-1a-3p and the sFRP1 3′-UTR binding sites. Luciferase activity in different groups after transfection. (e) The mRNA levels of sFRP1 after treatment with ox-LDL and ox-LDL + EPA (0 *μ*M, 50 *μ*M, 100 *μ*M, 200 *μ*M, 400 *μ*M, 600 *μ*M, and 800 *μ*M) (normalized to GAPDH) (*n* = 3 per group). (f) WB analyses of sFRP1, p-JNK, and JNK after ox-LDL treatment and NC/miR-1a-3p mimic/inhibitor transfection (*n* = 3 per group). (g) WB analyses of p-JNK and JNK after NC/miR-1a-3p mimic and sFRP1 NC/OE plasmid transfection (*n* = 3 per group). (h) The mRNA levels of sFRP1 after NC/miR-1a-3p mimic/inhibitor transfection on the basis of ox-LDL + 200 *μ*M EPA (*n* = 3 per group). (i) WB analyses of p-JNK and JNK after NC/miR-1a-3p mimic/inhibitor transfection on the basis of ox-LDL + 200 *μ*M EPA (*n* = 3 per group). The data are presented as the mean ± SEM. ^∗^*p* < 0.05,  ^∗∗^*p* < 0.01, and^∗∗∗^*p* < 0.001.

miRNAs mostly perform their roles by influencing the expression of their target genes. Therefore, our next step was to investigate the target genes of miR-1a-3p. The target genes of miR-1a-3p were predicted and intersected with the online tools TargetScan, miRanda, and microT-CDS. In total, there were 211 genes in the cross set (Figure 5(a)). As mentioned above, the Wnt pathway plays an essential role during treatment with EPA. Among the intersecting target genes, sFRP1 piqued our interest.

As expected, the mRNA level of sFRP1 was downregulated by miR-1a-3p mimics, while its level was upregulated by miR-1a-3p inhibitors (Figure 5(b)). Additionally, the sFRP1 protein level was further verified (Figure 5(c)). Then, the dual-luciferase reporter assay showed that sFRP1 reduced luciferase activity (Figure 5(d)). These results suggested that miR-1a-3p can regulate the expression of sFRP1. Then, we explored the expression of sFRP1 in ox-LDL-treated cells. qPCR analysis showed that after incubation with ox-LDL, the sFRP1 level decreased, while EPA treatment increased the sFRP1 level. When the concentration of EPA increased to 200 *μ*M, the mRNA level of sFRP1 was its highest (Figure 5(e)).

sFRP1 is an important Wnt antagonist. The Wnt pathway modulates a diverse range of cellular activities, including inflammatory responses [29, 30]. Since the Wnt/PCP-JNK pathway is closely associated with lipid metabolism, inflammation, and cell proliferation [31] and sFRP1 is known to regulate Wnt/PCP-JNK in cardiac myoblasts [32], we explored whether miR-1a-3p regulated the inflammation induced by atherosclerosis via sFRP1/Wnt/PCP-JNK signaling.

The levels of sFRP1, p-JNK, and JNK were then detected in ox-LDL-induced models. In ox-LDL-induced cells, miR-1a-3p mimics suppressed the expression of sFRP1 and augmented the activation of Wnt/PCP-JNK signaling. Reciprocally, miR-1a-3p inhibitors promoted the expression of sFRP1 and attenuated pathway activation (Figure 5(f)). Next, we explored the effects of miR-1a-3p and sFRP1 intervention on Wnt/PCP-JNK pathway. With the incubation of ox-LDL, miR-1a-3p could further activate JNK pathway while the transfection of sFRP1 plasmid could offset the trend. These results suggest that miR-1a-3p modifies the activation of JNK pathway via sFRP1 (Figure 5(g)). Based on the results above, we think miR-1a-3p participates in the process of atherosclerosis by targeting the sFRP1/Wnt/PCP-JNK axis.

Moreover, we detect the conditions under EPA treatment. RAW264.7 cells incubated with ox-LDL downregulated the level of sFRP1 and increased the p-JNK level and ratio of p-JNK/JNK, while EPA offset these trends. miR-1a-3p mimics abolished the EPA-induced upregulation of sFRP1 and amplified the EPA-induced activation of the JNK axis; miR-1a-3p inhibitors showed the opposite effects (Figures 5(h) and 5(i)). All of the above results suggest that EPA affects ox-LDL-induced RAW264.7 cells through the miR-1a-3p/sFRP1/Wnt/PCP-JNK axis.

**Figure 6:**
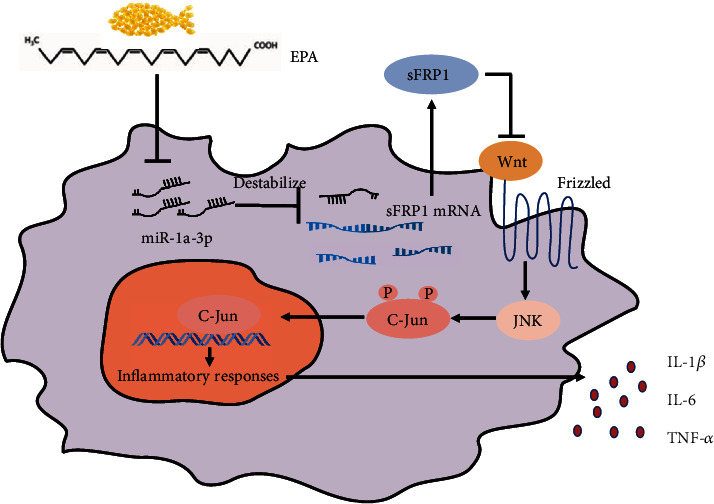
A schematic diagram of the effects of EPA in macrophages. In ox-LDL-induced foam cells, EPA suppressed miR-1a-3p activity, thus increasing the expression of sFRP1, which downregulated the activation of the Wnt/PCP-JNK axis. Therefore, inflammatory responses and oxidative stress were suppressed in atherosclerosis.

In conclusion, these results indicate that EPA ameliorates the inflammatory response and oxidative stress in atherosclerosis via the miR-1a-3p/sFRP1/Wnt/PCP-JNK axis (Figure 6).

## 4. Discussion

Previous studies have shown that increasing the purity of EPA can improve its anti-inflammatory effects in macrophages [33]. In the current study, we demonstrated the effects and mechanism of highly purified EPA both *in vivo* and *in vitro*. EPA significantly reduced plaque accumulation and lowered plasma lipid levels and inflammation levels in atherosclerotic mice. These phenomena were also be observed in macrophages *in vitro*. To explore the mechanism, microRNA sequencing and bioinformatics analyses were performed, and miR-1a-3p was found to have changed the most. Previously, miR-1a-3p was reported to play a role in oxidative stress in a hypoxia/reoxygenation model through the miR-1a-3p/glucose regulated protein 94 pathway [27] and affect metabolism in muscles and visceral adipocytes [34]. Statin, a classical medicine used in atherosclerosis, was suggested to cause skeletal injury through miR-1a-3p/mitogen activated protein kinase kinase kinase 1 [35]. Therefore, we first explored the effects of miR-1a-3p in inflammation process during atherosclerosis. A dual-luciferase reporter system confirmed that miR-1a-3p influenced the expression of sFRP1, which is an antagonist of the Wnt pathway and was previously indicated to negatively regulate Wnt/PCP-JNK signaling [32].

There has been detailed research on the sFRP family in cardiovascular diseases. sFRP1 was found to be a novel anti-inflammatory factor that acts on neutrophils after myocardial infarction [36]. sFRP4 takes part in ROS processes [37]. sFRP5 acts as an anti-inflammatory mediator in atherosclerosis and works through the Wnt pathway [38]. Therefore, we explored the effects of sFRP1 on inflammation and oxidative responses. Although there has been research on sFRP1 and the Wnt/PCP-JNK pathway in cardiomyocytes during cardiac injury [32], research on sFRP1 and Wnt/PCP-JNK in macrophages during atherosclerosis is novel.

Wnt is an ancient and evolutionarily conserved pathway that participates in several physiological processes, such as lipid modification, cellular interactions, inflammation, cell proliferation, and cell migration [39]. We first proved that miR-1a-3p modulated sFRP1/Wnt/PCP-JNK signaling. This signaling axis has been linked to oxidative stress, metabolic dysfunction, and inflammatory responses, which is consistent with our research [40].

There has yet to be research on the mechanism of highly purified EPA in atherosclerosis. After the Japan EPA Lipid Intervention Study (JELIS) showed that EPA could significantly lower the risks of coronary events [41], several trials showed negative results [42–46] until the REDUCE-IT trial showed that highly purified EPA could reduce the risk of ischemic events and cardiovascular death in patients with hyperlipidemia despite the use of statins [3]. The dose and purity of EPA have differed in clinical trials, which may explain the discrepancies in the results. Recently, a study showed that the additional risk reduction in REDUCE-IT trial was due to EPA and placebo [47], highlighting the importance of probing EPA's mechanism of action. In our research, we prepared highly purified EPA, excluding disturbances from impurities. The gas chromatography results showed that our material was almost entirely unsaturated fatty acids, adding to the validity of our study. The dose of EPA given to mice was based on that administered in the REDUCE-IT trial. Besides, PBS was used as placebo, more convincing than mineral oil in REDUCE-IT trial. On this basis, we found that EPA influenced the miR-1a-3p/sFRP1/Wnt/PCP-JNK axis, providing useful evidence for the clinical promotion of EPA.

EPA strongly ameliorated the progression of atherosclerosis in a murine model. The smaller plaque areas, lower levels of inflammation in the aortic roots and lower circulating levels of MCP-1 suggested that EPA strongly reduced the inflammatory response in the aorta. This phenomenon may explain the positive results from the REDUCE-IT trial. In this trial, the circulating levels of highly sensitive C-reactive protein in the highly purified EPA group were significantly lower than those in the placebo group [3], which is consistent with our results. In addition, highly purified EPA significantly reduced the rates of the primary endpoints, including nonfatal myocardial infarction, nonfatal stroke, coronary revascularization, and unstable angina. The Canakinumab Anti-inflammatory Thrombosis Outcome Study (CANTOS), another multicenter placebo-controlled trial, proved that IL-1*β* targeted therapy can prevent recurrent cardiovascular events to some extent [48]. Both trials proved that anti-inflammation interventions had a profound impact on cardiovascular events. In the future, we may explore the effects and mechanism of EPA in other cardiovascular diseases.

A limitation of this research is that we explored only miR-1a-3p/sFRP1/Wnt/PCP-JNK in macrophages. Since sFRP1 is a secreted protein and macrophage activation is only a driving factor of atherosclerosis, sFRP1 can act on other adjacent cells, such as endothelial cells and vascular smooth muscle cells; thus, research on this topic is ongoing. In addition, conservation between species was not identified. We tested the effects of EPA in mice only, which is less convincing. However, information from different online prediction tools showed that the potential binding sites of miR-1a-3p were conserved, which may be suggestive for clinical scenarios.

Overall, the anti-inflammatory effects of EPA were identified both *in vitro* and *in vivo*. First, we proved that EPA acts as an important anti-inflammatory regulator via miR-1a-3p/sFRP1/Wnt/PCP-JNK signaling in macrophages. As the initiator and amplifier of inflammatory responses in atherosclerosis, macrophages treated with EPA showed decreased levels of inflammation and the downregulation of oxidative stress, elucidating the mechanism of EPA. To summarize, the application of EPA in clinical practice is feasible.

## Data Availability

The data used to support the findings of this study are available from the corresponding author upon request.

## Supplementary Materials

Supplementary 1 Figure S1: EPA has a trend to upregulate the serological level of SOD in atherosclerosis mice (*n* = 5 per group).

Supplementary 2 Figure S2: the transfection of miR-1a-3p mimics and inhibitors did not influence the level of SOD (*n* = 3 per group).

Supplementary 3 Figure S3: the effects of interference of miR-1306-3p in macrophage lipid accumulation. Representative images of ORO staining and the percentage of foam cells, showing that miR-1306-3p mediated foam cell formation. Approximately 1000 cells were counted per treatment over six separate experiments.

Supplementary 4 Table S1: sequences of PCR primers. The sequences of primers used in the research are listed.

Supplementary 5 Table S2: analysis of fatty acid composition of EPA of different purity. The gas chromatography results of the EPA used in research, low-purity fish oil softgels, and high-purity fish oil softgels are listed in the table.
