# Supplementary figures and images for: Highly Purified Eicosapentaenoic Acid Alleviates the Inflammatory Response and Oxidative Stress in Macrophages during Atherosclerosis via the miR-1a-3p/sFRP1/Wnt/PCP-JNK Pathway
Source: Oxid Med Cell Longev. 2022 Apr 13;2022:9451058. doi: 10.1155/2022/9451058 (PMC9021996; doi:10.1155/2022/9451058)

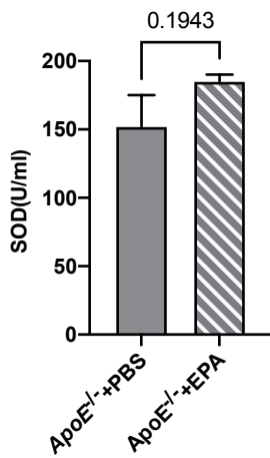

Supplement: Supplementary 1 — Figure S1: EPA has a trend to upregulate the serological level of SOD in atherosclerosis mice (n = 5 per group). [file 9451058.f1.pdf]

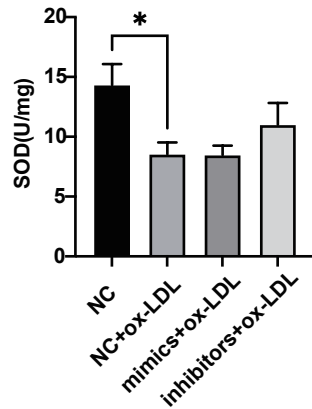

Supplement: Supplementary 2 — Figure S2: the transfection of miR-1a-3p mimics and inhibitors did not influence the level of SOD (n = 3 per group). [file 9451058.f2.pdf]

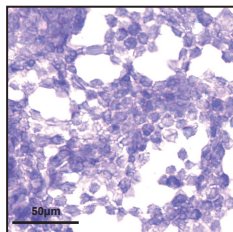

NC

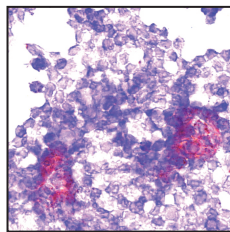

NC+ox-LDL

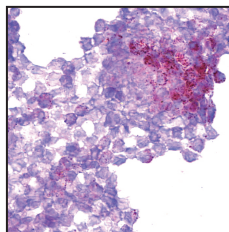

mimics+ox-LDL

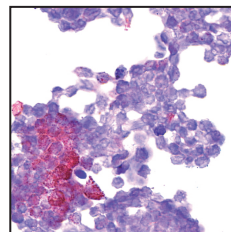

inhibitors+ox-LDL

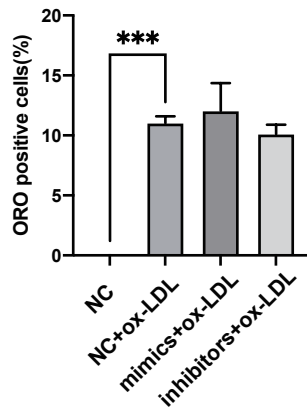

Supplement: Supplementary 3 — Figure S3: the effects of interference of miR-1306-3p in macrophage lipid accumulation. Representative images of ORO staining and the percentage of foam cells, showing that miR-1306-3p mediated foam cell formation. Approximately 1000 cells were counted per treatment over six separate experiments. [file 9451058.f3.pdf]
