# Supplementary material for: Highly Purified Eicosapentaenoic Acid Alleviates the Inflammatory Response and Oxidative Stress in Macrophages during Atherosclerosis via the miR-1a-3p/sFRP1/Wnt/PCP-JNK Pathway
Source: Oxid Med Cell Longev. 2022 Apr 13;2022:9451058. doi: 10.1155/2022/9451058 (PMC9021996; doi:10.1155/2022/9451058)
Supplement: Supplementary 4 — Table S1: sequences of PCR primers. The sequences of primers used in the research are listed. [file 9451058.f4.docx]

Table S1. Sequences of PCR primers.

| Gene/microRNA | Sequences |
| --- | --- |
| mmu-miR-1a-3p | F: 5’-TGGAATGTAAAGAAGTATGTAT-3’ |
| mmu-miR-1306-3p | F: 5’-ACGTTGGCTCTGGTGGTGATG-3’ |
| mmu-miR-194-5p | F: :5’-TGTAACAGCAACTCCATGTGGA-3’ |
| mmu-miR-582-3p | F: 5’-TAACCTGTTGAACAACTGAAC-3’ |
| U6 | F: 5’- CAGCACAAAAGGAAACTCACC-3’ |
| IL-1β | F: 5’ -TGGACCTTCCAGGATGAGGACA-3’  R: 5’ -GTTCATCTCGGAGCCTGTAGTG-3’ |
| TNF-α | F: 5’ -GGTGCCTATGTCTCAGCCTCTT-3’  R: 5’ -GCCATAGAACTGATGAGAGGGAG-3’ |
| sFRP1 | F: 5’ - GAGATGGTTTCAAAGGCTAACT-3’  R: 5’ - CAGGGACAACAGGAGTAAGG-3’ |
| GAPDH | F: 5’ -CATCACTGCCACCCAGAAGACTG-3’  R: 5’ -ATGCCAGTGAGCTTCCCGTTCAG-3’ |
