# Supplementary material for: Highly Purified Eicosapentaenoic Acid Alleviates the Inflammatory Response and Oxidative Stress in Macrophages during Atherosclerosis via the miR-1a-3p/sFRP1/Wnt/PCP-JNK Pathway
Source: Oxid Med Cell Longev. 2022 Apr 13;2022:9451058. doi: 10.1155/2022/9451058 (PMC9021996; doi:10.1155/2022/9451058)
Supplement: Supplementary 5 — Table S2: analysis of fatty acid composition of EPA of different purity. The gas chromatography results of the EPA used in research, low-purity fish oil softgels, and high-purity fish oil softgels are listed in the table. [file 9451058.f5.docx]

Table S2. Analysis of fatty acid composition of EPA of different purity

| Fatty Acid Compositions | Low-Purity Fish oil Softgels | High-Purity Fish Oil Softgels | 97% EPA |
| --- | --- | --- | --- |
| C12:0 | 0.1 | ND | ND |
| C14:0 | 8.5 | 0.5 | ND |
| C15:0 | 0.6 | ND | ND |
| C16:0 | 19.2 | 0.7 | ND |
| C16:1 | 10.4 | 0.4 | ND |
| C17:0 | 0.6 | ND | ND |
| C18:0 | 4.0 | 4.8 | ND |
| C18:1 | 8.8 | 7.2 | ND |
| C18:1t | 0.2 | 0.2 | ND |
| C18:2 | 4.6 | 0.9 | ND |
| C18:2t | 0.4 | 0.3 | ND |
| C18:3（ALA） | 1.3 | 1.5 | 0.6 |
| C18:3（GLA） | 0.3 | 0.1 | 0.2 |
| C20:0 | 0.3 | 0.9 | ND |
| C20:1 | 1.4 | 3.0 | ND |
| C20:2 | 0.5 | 0.7 | ND |
| C20:3 | ND | 0.3 | ND |
| C20:3 | 0.2 | 0.4 | ND |
| C20:5（EPA） | 21.3 | 45.1 | 99 |
| C21:0 | 0.1 | 0.2 | ND |
| C22:0 | 0.2 | 0.3 | ND |
| C22:1 | 0.2 | 0.3 | ND |
| C22:2 | ND | 0.1 | ND |
| C22:6（DHA） | 14.7 | 29.6 | ND |
| C20:4（ARA） | 1.2 | 1.9 | ND |
| C24:1 | 0.6 | 0.4 | ND |

Notes: ALA=α-linolenic acid; GLA=γ- linolenic acid; EPA= eicosapentaenoic acid; DHA=docosahexaenoic acid; ARA= Arachidonic acid; ND=not detected. The detection limit was 0.1%.
